# Endocrine Correlates of Musth in Free-Ranging Asian Elephants (*Elephas maximus*) Determined by Non-Invasive Faecal Steroid Hormone Metabolite Measurements

**DOI:** 10.1371/journal.pone.0084787

**Published:** 2013-12-17

**Authors:** Ratna Ghosal, André Ganswindt, Polani B. Seshagiri, Raman Sukumar

**Affiliations:** 1 Centre for Ecological Sciences, Indian Institute of Science, Bangalore, India; 2 Endocrine Research Laboratory, Department of Anatomy and Physiology, Faculty of Veterinary Science, University of Pretoria, Onderstepoort, Republic of South Africa; 3 Mammal Research Institute, Department of Zoology and Entomology, University of Pretoria, Pretoria, Republic of South Africa; 4 Department of Molecular Reproduction, Development and Genetics, Indian Institute of Science, Bangalore, India; Max Planck Institute for Evolutionary Anthropology, Germany

## Abstract

The occurrence of musth, a period of elevated levels of androgens and heightened sexual activity, has been well documented for the male Asian elephant (*Elephas maximus*). However, the relationship between androgen-dependent musth and adrenocortical function in this species is unclear. The current study is the first assessment of testicular and adrenocortical function in free-ranging male Asian elephants by measuring levels of testosterone (androgen) and cortisol (glucocorticoid – a physiological indicator of stress) metabolites in faeces. During musth, males expectedly showed significant elevation in faecal testosterone metabolite levels. Interestingly, glucocorticoid metabolite concentrations remained unchanged between musth and non-musth periods. This observation is contrary to that observed with wild and captive African elephant bulls and captive Asian bull elephants. Our results show that musth may not necessarily represent a stressful condition in free-ranging male Asian elephants.

## Introduction

Sexual activity in mature male elephants is predominantly associated with the occurrence of musth [1–3], a phenomenon well documented in the Asian elephant (*Elephas maximus*) since ancient times [4]. In the early 1980s, musth has also been recognized in male African elephants *Loxodonta africana* [3]. A prominent visual characteristic in African as well as in Asian musth bulls is the secretion from an active pair of temporal glands [3,5-7]. When entering into musth, elephant bulls also often show a greenish discoloration of the penis sheath, as well as a continuous discharge of urine (urine dribbling), which has a typical strong odour [1-3,5,6]. Among African elephants, musth periods are usually also associated with an increase in aggression, dominance displays and unpredictability, especially towards other bulls in musth [3,8]. Similarly, musth may be the most important determinant of dominance among male Asian elephants [9] and this may signal to females a “handicap” [10] through its burden on the animal’s immune system [11]. 

 Several studies have demonstrated a distinct musth-related elevation in androgen levels in male African elephants in captive and wild settings, as well as for zoo-housed and working Asian elephant bulls [1,2,12-15]. In contrast, the relationship between musth and glucocorticoid hormones (indicator of stress) remains unclear. Studies on captive and wild African elephant bulls showed reduced faecal glucocorticoid metabolite levels during musth in comparison to non-musth phase, indicating that musth is a not necessarily a stressful condition for males [16,17]. However, Brown et al. [18] and Yon et al. [19] found a moderate but significant positive correlation between circulating plasma testosterone and cortisol levels for captive African and Asian bulls in musth, thus supporting the hypothesis that musth may represent a physiologically stressful condition. The main technical difference between the two studies was the use of circulatory [18,19] versus excreted steroids [16,17]. Further studies relying on simultaneous measurements of circulatory and excreted steroids may be necessary to resolve that discrepancy. So far, however, no information on musth and associated changes in glucocorticoid and testicular activity is available for free-ranging Asian elephants. The current study, therefore, is aimed at providing basic information on the musth-related endocrine status of free-ranging Asian elephants which may help to clarify the relationship of musth and adrenocortical function.

 In contrast to the African elephant, most of the studies reporting on endocrine measurements in male Asian elephants are based on determining hormone levels in either plasma or serum [1,2,18,19]. To understand the hormonal correlates of musth in wild Asian bull elephants, a non-invasive approach for endocrine assessment is mandatory, as blood sampling from free-ranging animals would not be feasible or even desirable for an endangered species. In this respect, the collection of urine or faeces for endocrine analysis is a reliable alternative to blood hormone measurements, demonstrated by a large number of established non-invasive methods for monitoring gonadal as well as adrenocortical function in a variety of mammalian species [20–23], including the African [13,24] and Asian elephant [25–27]. Thus, the overall aim of this study was to describe the endocrine correlates of musth in wild male Asian elephants based on non-invasive assessment of testicular (androgen) and adrenocortical (glucocorticoid) function.

## Materials and Methods

### Study area and elephant population

Field work was conducted over a period of about three months (April-June 2011) in Kaziranga National Park, Assam, India. The park sustains an elephant population with a density of about 1.2 individuals/km^2^ over an area of 993 km^2^ (Kaziranga National Park census report, 2011). Located in the floodplains of the Brahmaputra, the park experiences an annual rainfall of 1500-2500 mm along a west-east gradient and features patches of tropical semi-evergreen forests amidst extensive tall and short grassland surrounding numerous water bodies [28]. Data were collected in three ranges of the national park, namely, Agratoli (eastern range), Kohora (central range) and Bagori (western range).

### Sampled individuals

In total 77 faecal samples were collected from 60 adult male elephants, assigned to either be in musth (44 samples from 27 males) or in non-musth (33 samples from 33 males) (Table 1). Multiple samples (range 2-7) were collected from nine bulls in musth.

**Table 1:** Number and categorisation of faecal samples collected from individual free-ranging Asian elephant bulls in Kaziranga, India.

**Category**	**No. of males**	**No. of samples**
**Non-musth**	**33**	**33**
**Musth (TGS and/or UD)**	**27**	**44**
**Total**	**60**	**77**

TGS indicates temporal gland secretions and UD indicates urine dribbling.

### Morphological parameters

 The adult (>20yr) male elephants were recorded using a Sony Handycam (HDR-SR10E). Each male was further photographed and characteristic morphological features such as ear-fold, tail characteristics, and pigmentation pattern noted. For each bull, the presence and degree of two characteristic musth signs, namely temporal gland secretion (TGS) and urine dribbling (UD) were recorded on the day of the faecal sample collection. Visual estimation of shoulder height was used as a criterion to determine the age of the observed males [29]. 

 Musth bulls showed distinct differences in the occurrence and degree of the two monitored physical signs of musth (TGS and UD), with variation seen in an individual male observed on consecutive days or even during a single day. Because of this variation in physical signals of musth, the faecal samples were grouped into only two categories – from males in musth (TGS and/or UD present) and from males in non-musth (absence of both TGS and UD) [18,19]. 

### Faecal sample collection

Approximately 100-250 g of feces was taken from the middle of a bolus shortly after a focal animal had defecated and moved away. Aliquots of each faecal sample were stored at -20°C at the field site until transported on ice to the Indian Institute of Science, Bangalore, for analysis. Permits and approvals were obtained for the work from the Forest Department of Assam, India.

### Sample extraction and hormonal assays

Extraction and analysis of steroid metabolites were carried out according to Ganswindt et al. [17]. In brief, frozen faecal samples were lyophilized, pulverised, and sifted using a nylon mesh strainer to remove fibrous material [17].

 Approximately 50 mg of faecal powder was extracted using 3ml of 80% ethanol. After vortexing for 15 min., the mixture was centrifuged at 1500 g for 10 min. at 37°C. The supernatant was then decanted into microcentrifuge tubes and stored at -20°C until analysis.

 The resulting extracts were measured for immunoreactive androgen metabolites using an epiandrosterone enzyme immunoassay (EIA) for faecal androgen metabolites (FAM), which has been shown to be potentially useful for monitoring male reproductive function in several mammalian species [30,31] including African and Asian elephants [13,17,32]. To measure faecal glucocorticoid metabolites (FGM), an 11-oxoaetiocholanolone EIA detecting FGM with a 5β-3α-ol-11-one structure was used. This EIA has previously been shown to provide reliable information on adrenocortical function in African as well as captive male Asian elephants [24,26,33]. The FAM assay used an antibody against 5α-androstane-3α-ol-17-one-HS and 5α-androstane- 3,17-dione-thioether conjugated with biotin as a label [30]. The FGM EIA used an antibody against 5β-androstane-3α-ol-11-one-17CMO:BSA and 5β-androstane-3α-ol-11,17-dione-17-CMO-biotinyl-3,6,9 trioxaundecanediamin as a label [32]. Cross-reactivities of the two antibodies are described in Ganswindt et al. [13] for FAM and in Möstl et al. [34] and Huber et al. [35] for FGM. 

Sensitivity of the two assays at 90% binding was 1.9 pg/well for FAM and 0.9 pg/well for FGM, respectively. Intra- and inter-assay coefficients of variation, determined by repeated measurements of high and low value quality controls, respectively, ranged between 7.3% and 14.7% (for FAM) and 4.2% and 13.8% (for FGM). 

### Data Analysis

Based on the available photos and video footage, only one sampled elephant was clearly common to both the musth and non-musth categories. All samples from this animal were removed from both the categories (musth and non-musth), prior to statistical analysis. Mean hormone values were calculated for those males for which multiple samples were present in the musth category, and used in subsequent analyses. Data sets were log-transformed and examined for normality using Shapiro-Wilks test (p>0.1 for both FAM and FGM categories) and equal variance using F-test (FAM: F_(cal)_ 3.63, P < 0.001; FGM: F_(cal)_ 5.16, P < 0.001); subsequently, differences in faecal hormone concentrations between two sets (musth and non-musth) of data were examined by two tailed unpaired t-test for unequal variances (Aspin-Welch). The α level of significance was set at 0.05. The computer program Ky Plot 2.0 was used for all statistical analyses. 

## Results

**Figure 1:**
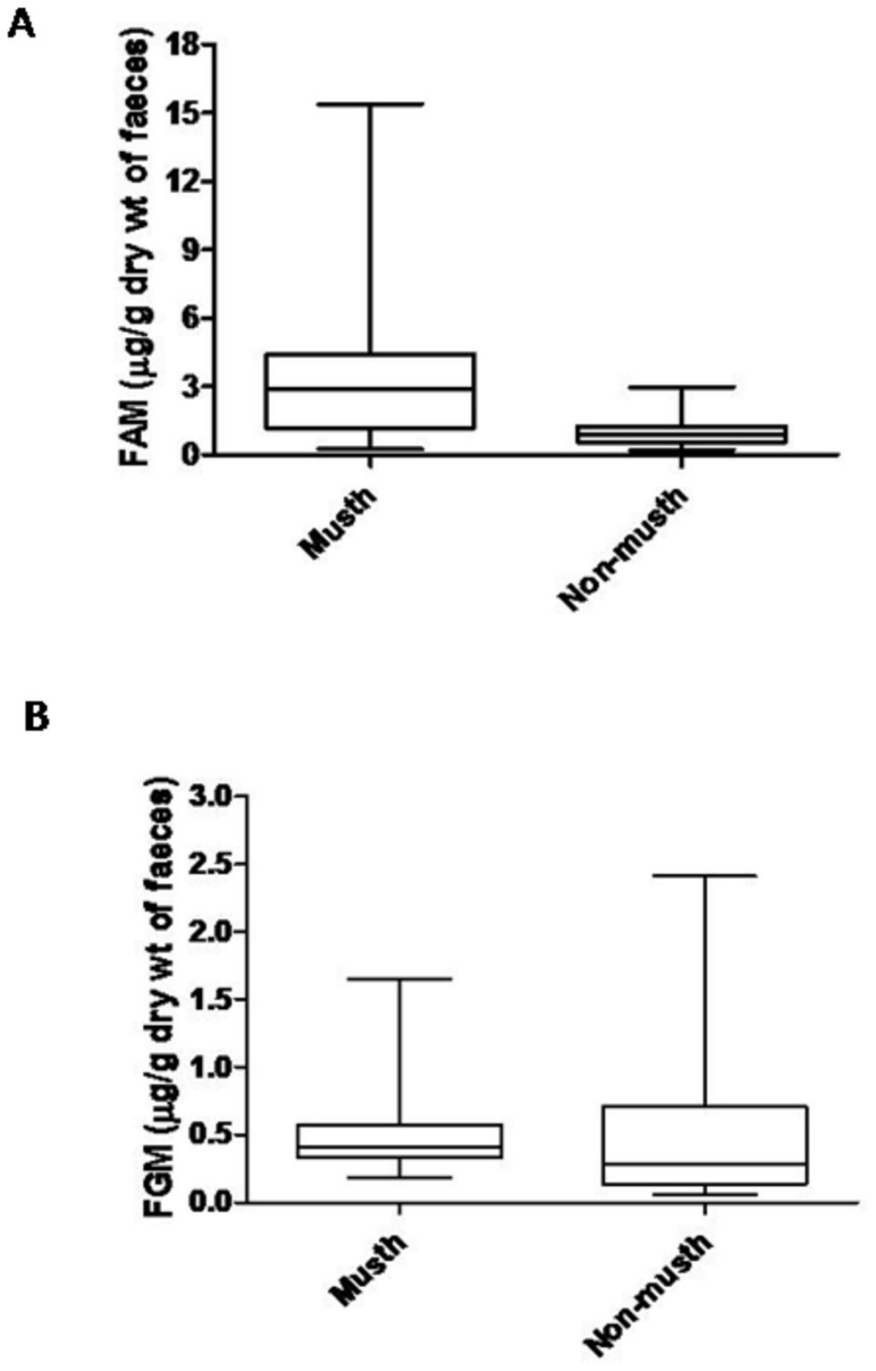
Hormonal data of adult Asian bull elephants in Kaziranga, India. Faecal epiandrosterone metabolite (FAM) (A) and faecal glucocorticoid metabolite (FGM) (B) levels of 60 adult bulls during musth (n = 27) and non-musth (n = 33) conditions. The asterisk indicates statistically significant difference (p<0.001) between chosen categories.

Over the 3-month field study, a total of 60 bulls were individually identified. Out of the 60 males, 27 were assigned to be in musth based on their physical signs (TGS and/or UD). For bulls in musth, immunoreactive FAM levels varied between 0.24 and 15.38 µg/g dry weight and FGM concentrations ranged between 0.19 to 1.65 µg/g dry weight. Non-musth males showed FAM and FGM levels in the range of 0.20 - 2.96 µg/g dry weight and 0.06 - 2.41 µg/g dry weight, respectively. FAM levels of bulls showing physical signs of musth were significantly higher (t = 4.3, p < 0.001) compared to non-musth males (Figure 1a). In contrast, immunoreactive FGM levels were not significantly different between the two categories (t = 1.61, p > 0.05) (Figure 1b). 

## Discussion

Our observations of a population of free-ranging Asian elephants in north-eastern India show that musth bulls have comparatively higher FAM levels than do non-musth males (Figure 1a), a finding consistent with all earlier published data on plasma testosterone levels in captive Asian elephants [1,18,19] as well as on captive and free-ranging African elephants [14,17]. These results, therefore, demonstrate that the described FAM assay is a useful non-invasive tool for assessing testicular endocrine function in free-ranging Asian elephant bulls.

 In contrast to studies on captive Asian elephants [18,19] and on captive and free-ranging African elephants [14,16,17,24], our data show that musth does not seem to be associated with changes in FGM levels in free-ranging Asian elephants (Figure 1b). The difference in the results between the current study on free-ranging Asian elephants and previous studies on captive Asian elephants [18,19] can be attributed to several factors such as environmental conditions (captive versus free-ranging), measurement of circulating versus excreted steroids (also indicated by Brown et al. [18]), and differences in the applied immunoassay procedures using diverse antibody specificities for quantification. The current study on free-ranging Asian elephants closely supports the findings of Rasmussen et al. [15] that also relied on cross-sectional sampling of faeces from free-ranging African elephants and reported unchanged levels of FGM in relation to the occurrence of musth. However, studies based on a larger data set and longitudinal sampling of captive and wild African elephants by Ganswindt et al. [14,16,17] showed reduced levels of FGM during musth phase when compared to the non-musth phase of the same individuals. A negative correlation between musth and stress levels in the African species [14,16,17,24] was explained by a possible negative feedback mechanism between the hypothalamo-pituitary-gonadal (HPG) and hypothalamo-pituitary-adrenal (HPA) axes, as described in other mammals such as rodents [36]. This emphasizes the need to carry out longitudinal sampling at shorter periodic intervals on free-ranging Asian elephants that may provide better insights into the association between the HPA and HPG axes regulation of musth.

 The nature of the cross-sectional data was also reflected in the variation in FAM levels during the musth period (Figure 1a). Such variation may be due to different stages of musth (TGS+UD present versus only TGS present) (Table 1) and/or different age groups among the individuals sampled. However, the observed variation in FAM levels in African elephants was attributed to different musth stages rather than the age of the individuals sampled [14]. Variability of FGM levels in both the musth and non-musth categories (Figure 1b) probably indicates the role of other ecological/environmental factors as opposed to the sexually-active musth phase contributing to such variation [15]. Such variation in FGM levels (Figure 1b) may presumably also lead to non-significant differences between the two categories (musth and non-musth), as observed in our results. Moreover, confirmation that the measurements obtained with our FGM assay do, in fact, reflect adrenal glucocorticoid output is provided by the different musth-related patterns of FAM and FGM levels found (Figure 1). 

 Elevated levels of FGM as reported in earlier studies [18,19] of captive Asian elephants are most likely to be an imposed condition resulting from restraint in captivity. Numerous anecdotal accounts and observations on the behaviour of captive bull elephants clearly suggest that they may become aggressive and uncontrollable during the musth phase as they attempt to break free of their restrained condition [37,38]; such captive bulls in musth are presumably exhibiting their natural behaviour of wandering more widely in search of estrous females for mating [39]. On the other hand, free-ranging Asian bull elephants do not necessarily show overt signs of aggression when they come into musth and are dominant over non-musth bulls [9]. Thus, levels of stress in free-ranging bull elephants in musth could be expected to be lower than those of bulls in musth that have been restrained under captive conditions. 

 In conclusion, this study is the first report on endocrine correlates of musth in wild Asian elephants using non-invasive measurement of FAM and FGM. Elevated levels of FAM during the musth phase are in accordance with earlier studies indicating the role of androgens during the sexually-active period of male elephants. Based on the pattern of FGM, however, our study shows that musth in wild Asian elephant bulls does not necessarily represent a physiological stress associated with the activation of hypothalamic-pituitary-adrenal axis. Completely contrasting environmental conditions between captive and free-ranging bulls may contribute towards the observed differences in musth-related glucocorticoid activity. In this regard, longitudinal studies might help to better understand the relationship between occurring physical signs of musth and their endocrine correlates in free-ranging Asian elephant bulls.
